# Prevalence of and risk factors associated with depression among nursing students acting on the frontline of COVID-19 pandemic: A cross-sectional study

**DOI:** 10.3389/fpubh.2022.1020419

**Published:** 2023-01-09

**Authors:** Huyen Thi Hoa Nguyen, Anh Phuong Hoang, Linh Manh Vu, Duc Quang Tran, Linh Khanh Bui, Thuan Thi Pham, Linh Thuy Khanh Tran, Huong Thi Thanh Nguyen

**Affiliations:** ^1^College of Health Sciences, VinUniversity, Hanoi, Vietnam; ^2^Faculty of Nursing and Midwifery, Hanoi Medical University, Hanoi, Vietnam; ^3^Department of Nursing, Military Institute of Traditional Medicine, Hanoi, Vietnam; ^4^Faculty of Nursing and Medical Technology, Ho Chi Minh City Medicine and Pharmacy University, Ho Chi Minh City, Vietnam

**Keywords:** depression, nursing students, Vietnam, pandemic, mental health, COVID-19

## Abstract

**Introduction:**

The widespread devastation caused by the ongoing waves of COVID-19 imposed a significant burden on the healthcare labor force. At the frontline in the battle against the deadly COVID-19 virus, nursing students in Vietnam were at a much-increased risk of developing mental health conditions. This study aims to identify the prevalence of depression and its related factors, along with coping strategies used by nursing students in the COVID-19 pandemic in Vietnam.

**Materials and methods:**

The study was cross-sectional in nature, with convenient sampling at the epicenters of COVID-19 outbreaks in Vietnam (*N* = 191) from April to November 2021. After conducting a questionnaire pilot, the data was collected strictly using an internet-based approach. The Depression, Anxiety, and Stress Scale-21 items were used to identify the risk of depression among nursing students. The Chi-square test was used to assess the differences between coping strategies among nursing students. A multivariate logistic regression model was used to identify risk factors associated with depression.

**Findings:**

The percentage of nursing students affected by depression was 21.5%, and almost half of the nursing students (49.2%) had no coping strategies for dealing with mental health concerns. Among the remaining nursing students, video-based mental consultation was the most popular method (25.7%). Being females (AOR: 2.7, 95% CI: 1.1–6.7), collecting bio-samples (AOR: 2.9, 95% CI: 1.4–6.2), providing support to vaccination spots (AOR: 2.3, 95% CI: 1.1–5.1), and not vaccinating against COVID-19 (AOR: 3.1, 95% CI: 1.1–9.1) were found as risk factors for depression among nursing students.

**Conclusion:**

Our research revealed a significant number of nursing students suffering from depressive symptoms and underscoring the need for more effective methods of dealing with this condition. Depression management and coping skills focusing on female populations and those whose direct contacts with infectious sources should be implemented in the nursing curricula and continuous training credits. Those trainings, would support future nurses in handling crisis situations better.

## 1. Introduction

Nursing is considered to be one of the top first-line dedicated professions in disaster response (1), as well as any primary and secondary infectious disease prevention efforts, including COVID-19 (2, 3). During the COVID-19 pandemic, nurses need to reassure, inform, and support patients, patients' families, and the community to stay healthy following the latest guidance on COVID-19 prevention (4) while delivering the nursing care plan in all phases of the illness trajectory. In this harsh time, nurses are responsible for all patients' demands, including supplying and usage of sanitation materials and personal protective equipment, offering screening information, confinement guidelines, and triage or quarantine protocols (5). To be a health model and ensure all nursing tasks during the pandemic, nurses, therefore, must first be mentally and physically healthy. Conversely, nursing is considered as a high-risk group of occupational stress and a higher risk of depression than other groups of health professionals (3). The COVID-19 pandemic caused mental health concerns for nurses, becoming even more alarmed than other professionals as nurses continued to work while other residents stayed at home for their safety, making nurses more susceptible to the high risk of COVID-19 infection and mental disorders (6, 7). Evidence gathered over the previous years indicated a significant number of nurses suffering from depression, ranging from around 22.5 to 52%, according to several systematic studies (8–11).

The fourth wave of the COVID-19 pandemic in Vietnam started on 27th April 2021 and recorded 1,207,498 confirmed cases and 24.657 deaths until 28th November 2021 (12). Most positive cases were recorded from Ho Chi Minh, Binh Duong, and Dong Nai, mainly related to the Delta variant (13). The virus has spread in both the local communities and large industrial zones, putting a heavy burden on the whole healthcare system. In response to the Ministry of Health's call for assistance to fight COVID-19, many students from nursing schools voluntarily registered to be on the frontline fighting with COVID-19. In the previous waves in Vietnam, students' support has witnessed certain advantages in preventing and controlling the COVID-19 outbreak (14).

Like other countries, the continuous waves of COVID-19 placed a significant burden on the healthcare workforce in Vietnam. Hospitals, health centers, and clinics were always in an overloaded situation with continuous peaks in the numbers of suspected COVID-19 cases, positive cases, and ICU treatments, while the shortage of health workers could not be solved within months or weeks (14). To accommodate these difficult circumstances, nursing students and other healthcare students were called for their voluntary to participation in the “fence” against COVID-19 (4, 5). Vietnamese nursing students were treated as practice nurses under the supervision of lecturers and registered nurses to detect COVID-19 cases, support vaccination, and deliver caring activities. These emerged jobs and responsibilities with the boundary of COVID-19 infection fear placed them at high risk of mental illnesses. From the beginning of the COVID-19 pandemic, depression and associated mental health problems of frontline health workers have been examined in many papers in many other countries (15–17).

In developing countries as Vietnam where the future pandemic or latent diseases can affect the healthcare system and the whole community at any time, preparing for the future workforce is essential. Most publications in Vietnam typically evaluated the mental health of all health professionals instead of focusing on nurses, and none focusing on the special healthcare workforce as undergraduate nursing students who provided round-the-clock assistance to patients with the highest dedication and thoroughness during the pandemic (18–20). Only in this special scenario of the pandemic, the nursing students had the chance to act as a nurse with lots of ups and downs regarding the working experience and the emotional fluctuation. Therefore, this research aims to explore the prevalence of depression and its related factors and coping strategies used by nursing students in the COVID-19 pandemic in Vietnam. This will be one of the first papers providing evidence for nurse lecturers and nurse managers for their future working plans with nursing students during the pandemic as well as pinpointing useful predictors for socialists in their community mental health projects.

## 2. Materials and methods

### 2.1. Research design

A cross-sectional study design was used.

### 2.2. Research time and setting

The study was conducted in the hotspots of COVID-19 outbreaks in Vietnam from April to November 2021. Given the evidence that the prevalence of anxiety and depression among frontline healthcare workers was high during the COVID-19 pandemic, we employed a method known as purposive sampling, which involved selecting high-risk communities of COVID-19 pandemic from a comprehensive review of the material provided by the government. Finally, three large provinces and cities in the North and the South of Vietnam (Ho Chi Minh City, Binh Duong, and Hai Duong Province) were selected due to the most COVID-19 suffering regions of the country during that period (21). Therefore, these areas were the main settings to conduct the survey.

### 2.3. Research subjects

Nursing students acting as volunteers in both hospitals and the community on the front line at Hai Duong province, Binh Duong province, and Ho Chi Minh city were invited to participate in this study.

#### 2.3.1. The inclusion criteria

Nursing students who (1) were involved in supporting activities for suspected or confirmed COVID-19 patients either in the hospital or in the community; (2) worked in the frontline areas for at least 2 weeks; and (3) volunteered to take part in the research.

#### 2.3.2. The exclusion criteria

Nursing students who (1) did not involve in activities requiring directly contacted with suspected or confirmed COVID-19 patients; (2) have been diagnosed with mental disorders, or physical illness were excluded.

### 2.4. Sampling and process of data collection

#### 2.4.1. Sample size

Convenient sampling was employed. Nursing students who met the selection criteria were invited to participate in the study with a sample size calculated according to the formula provided by Lwanga and Lemeshow in 1991 (22):


$$\begin{array}{l} \frac{Z_{1-\alpha /2}^{2}\;p\left(1-p\right)}{d^{2}} \end{array}$$

α: Confidence level

p: Anticipate population proportion

d: Relative precision

n: Sample size

With α = 0.05, Z_1−α/2_ = 1.96, *p* = 0.52 according to prevalence of depression among nursing students in a systematic review assessed 17 studies (23), and *d* = 0.07, the estimated sample size was 196 participants. We added 10%, ensuring the required sample size if any attrition happened. The final sample size was 216.

#### 2.4.2. Data collecting process

The data was collected through an online-based method using Google Forms. Prior to the formal release of the online surveys, the research team piloted the questionnaires to ensure that the questions and instructions were clear and that any possibility for confusion was eliminated. The questionnaire was distributed to a group of around 10 nursing students in order to determine the time required to complete it and to ensure that the questions were clear and unambiguous. After contacting the nurse manager(s) of hospitals or community health stations at the hotspots of COVID-19 outbreaks for permission, a consent form and a link to the online questionnaire survey were directly sent to them for posting in the group-discussion forums/flat forms of those hospitals or health stations. Nursing students at the hotspots willing to participate in the study clicked the link (https://forms.gle/maZDkBFTmrJrVDAy6) and initially answered the questions relating to inclusion criteria. Once they met the study criteria, they were directly linked to a consent form and then a survey questionnaire for completion of participation. In addition to a set-up of single response, participation in the study was a voluntary basic, no incentive was provided. The data collected therefore were individual responses.

### 2.5. Research instruments

The research instrument is divided into 4 parts:

PART 1: Demographic information.

PART 2: The tool to assess the level of risk of depression of nursing students was 07 items of the Depression scale in the 21-item Depression Anxiety and Stress Scale (DASS-21, which were validated on rural northern Vietnamese women and translated into Vietnamese in 2013. The DASS21 was first translated from English into Vietnamese, then evaluated by a panel of health experts and research workers to ensure it was culturally and linguistically suitable, and then its document was translated back to English for final verification (24). The score was equaled by summing up the score of every question and then multiple two. The level of risk of depression among respondents will be “mild” if the total score from 10-13, “moderate” if the score from 14 to 20, “severe” if the score 21–27, and “very severe” if score higher than 28 up to 42.

PART 3: Tool to assess factors relating to the level of depression of nursing students was the questionnaire developed by Shechter and colleagues (25) with permission prior to data collection.

PART 4: Coping strategies, including questions about received, preferred, or applied strategies such as video-based approach, online consultation, online network, self-care to help nursing students deal with mental health problems.

### 2.6. Data management and analysis

SPSS version 26.0 was used to enter and analyze data. Data from the online survey was extracted and transferred to an SPSS file and then cleaned prior to analysis. If certain data were ambiguous or missing, the study team individually contacted individuals to get further information. All identified information of the study participants was removed prior to data analysis. Descriptive statistics were summarized and performed by tables and charts. The Chi-square test was used to examine the differences between coping strategies among nursing students. Binary logistics regression was performed to examine the influence of variables on perceived depression among nursing students, along with two-sided *p* < 0.05 was considered statistically significant. First, a bivariate analysis between perceived depression and its predictors was performed. Predictors showing a potential relationship with depression (*p* < 0.2) were selected to add to the regression model by using Enter method (26). No binary correlation between perceived depression and coping strategies was found. In the beginning, the model containing two independent variables (age and gender) was entered for the first step of the equation. Next, predictors including registered to be specifically on the current task, having family relatives working in the quarantine area, being vaccinated with COVID-19, collecting bio-samples in the community, supporting vaccination spots, and academic year were entered as a block for step 2 while controlling for the effects of age and gender. The Hosmer-Lemeshow test was used to determine the goodness of model fitting. The model outcomes were presented with adjusted OR (95%CI) after the entry of all independent variables.

## 3. Results

### 3.1. Demographic characteristics

A total of 216 nursing students voluntarily completed the online questionnaire. However, after cleaning the data and excluding ineligible responses, only 191 (88.4%) responses were included in the analysis. After checking against the margin of error which is vary from 5 to 10%, we found that the sample of 191 met requirements for power analysis and for reflecting the figure reported. Therefore, data analysis was performed on those 191 respondents.

Table 1 shows the characteristics of the study participants. Participants' age ranged from 19 to 25 years (*M* = 21.8; SD = 1.1). Of these 191 participants, 83.2% were women, and 99.0% were single. A large proportion (83.8%) of the nursing students who participated in the study do not have a history of chronic illness. Up to 96.3% of nursing students voluntarily selected their current working sides in epidemic prevention as frontline healthcare workers, while the rest registered their interest and then mobilized to be in the sides. About 65.4% of the study participants had family relatives working in the quarantine station, and only 89.0% of them were vaccinated with COVID-19. When asked about their primary task, about 67.0% collected bio-samples in the community. Most participants (85.9%) were university students, 44.0 and 33.5% of whom were year 3 and year 4 students, respectively.

**Table 1:** Characteristics of the study participants.

**Characteristics**		***n =* 191**	**Percentage (%)**
Age (Mean ± SD)		21.8 ± 1.1	
Gender	Male	32	16.8
	Female	159	83.2
Marital status	Engaged/living with partners	1	0.5
	Divorce/widowed	1	0.5
	Single	189	99.0
History of chronic illness	No	160	83.8
	Yes	31	16.2
Volunteer	To be on different tasks	7	3.7
	To be specifically on the current task	184	96.3
Had family relatives working in the quarantine station	Yes	125	65.4
	No	66	34.6
Being vaccinated with COVID-19	Yes	170	89.0
	No	21	11.0
Student's primary task on the frontline	Take care of confirmed positive cases	37	19.4
	Collect bio-samples in the community	128	67.0
	Follow up on suspected cases	26	13.6
	Contact tracing	41	21.5
	Epidemiological screening	40	20.9
	Volunteer at vaccination spots	71	37.2
	Administrative tasks and logistics	16	8.4
Level of education	University students	164	85.9
	College students	27	14.1
	Vocational students	0	0
Academic years	Year 1	3	1.6
	Year 2	40	20.9
	Year 3	84	44.0
	Year 4	64	33.5

### 3.2. The prevalence of perceived depression

**Figure 1:**
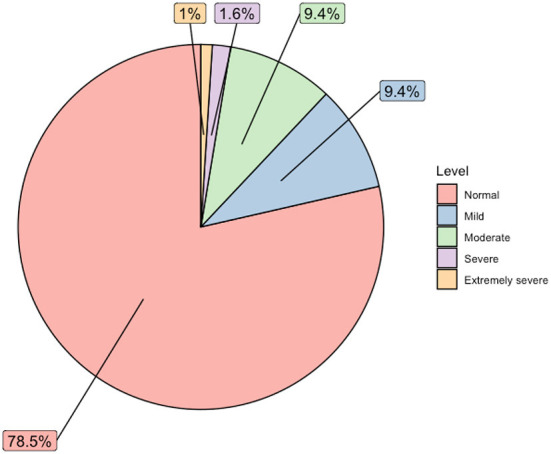
The prevalence of risk of depression among nursing students (*n* = 191).

As shown in Figure 1, the overall prevalence of mild-to-extremely severe levels of perceived depression among participants, was 21.5% (*n* = 41). In total, 12.0% (*n* = 23) of nursing students reported moderate-to-extremely severe perceived depression.

### 3.3. Strategies nursing students used to cope with depression

Participants were asked about the supporting method they received or applied by themselves to help them deal with mental health problems, including depression. Table 2 shows nearly half of the participants (49.2%) had no coping strategy for mental health issues. However, the rest of the nursing students also applied various supporting methods for themselves, among which the video-based approach of mental consultation was the most common, accounting for 25.7%; followed by partial support from professionals (25.1%) and an online network to support medical staff on the frontline (14.7%). In terms of coping strategies for depression, there was a significant association between depression with an online network supporting medical staff on the frontline (χ^2^ = 6.232; *p* < 0.05).

**Table 2:** Differences in perceived depression by coping strategy.

**Variables**	**Perceived depression**		**Total**	**Chi-square**	***p***

	**Yes (** ***n*** **, %)**	**No (** ***n*** **, %)**			
Total	41 (21.5)	150 (78.5)	191 (100)		
**Video-based approach**					
Yes	14 (28.6)	35 (71.4)	49 (25.7)	1.974	0.16
No	27 (19.0)	115 (81.0)	142 (74.3)		
**Group online consultation**					
Yes	2 (15.4)	11 (84.6)	13 (6.8)	0.306	0.738
No	39 (21.9)	139 (78.1)	178 (93.2)		
**Individual online- consultation**					
Yes	3 (15.8)	16 (84.2)	19 (9.9)	0.403	0.769
No	38 (22.1)	134 (77.9)	172 (90.1)		
**Online network to support medical staff on the frontline**					
Yes	1 (3.6)	27 (96.4)	28 (14.7)	**6.232**	**0.011**
No	40 (24.5)	123 (75.5)	163 (85.3)		
**Self-care for mental well-being with partial support from professionals**					
Yes	7 (14.6)	41 (85.4)	48 (25.1)	1.802	0.18
No	34 (23.8)	109 (76.2)	143 (74.9)		
**None of the above**					
Yes	22 (23.4)	72 (76.6)	94 (49.2)	0.412	0.521
No	19 (19.6)	78 (80.4)	97 (50.8)		

The bold values indicate the *p*-value less than 0.05 which is considered to be statistically significant in our results.

### 3.4. Factors influencing perceived depression among nursing students

In the first step, where only background factors, namely gender and age, were included in the primary model, only between 1.8% (Cox and Snell R square) and 2.8% (Nagelkerke R squared) of the variation of perceived depression was explained by these two factors. In the second step, other variables, including being vaccinated with COVID-19, collecting bio-samples in the community, and supporting vaccination spots, were entered and became significant predictors (*p* < 0.05). The complete model containing all predictors was statistically significant, χ^2^ (df = 9, *n* = 191) = 21.606, *p* < 0.001, indicating that the model could distinguish between students who reported and did not report depression problems. The model as a whole explained between 10.7 (Cox and Snell R square) and 16.5% (Nagelkerke R squared) of the variance in perceived depression and correctly classified 79.1% of cases. The Hosmer-Lemeshow goodness of fit indicated no evidence of poor fit with χ^2^ = 7.39, *p* = 0.389. As shown in Table 3, three independent variables made statistically significant contributed to the model (being vaccinated with COVID-19, collecting bio-samples in the community, and supporting vaccination spots). The strongest predictor of depression among nursing students was vaccinated with COVID-19, recording an odds ratio of 3.156. This indicated that respondents vaccinated were over 3 times more likely to perceive depression than those vaccinated against COVID-19 after controlling for all other factors in the model. Besides, nursing students potentially exposed to people at risk of COVID-19, specifically collecting bio-samples in the community and supporting vaccination spots, were over 2.9 and 2.3 times more likely to report depression than other primary tasks on the frontline.

**Table 3:** Predictors of depression among nursing students by binary logistic regression (*n* = 191).

**Block**	**Factors**	AOR^*^	***p***	**95%CI**	
**Block 1**	**Age**	0.997	0.984	0.729	1.363
	**Gender**				
	**Female**	2.257	0.06	0.965	5.278
	Male	Ref			
**Block 2**	**Age**	1.070	0.697	0.762	1.503
	**Gender**				
	Female	2.676	**0.036**	**1.068**	**6.705**
	Male	Ref			
	**Volunteer to be specifically on the current task**				
	Yes	4.259	0.094	0.78	23.248
	No	Ref			
	**Having family members on the frontline**				
	Yes	1.258	0.584	0.553	2.864
	No	Ref			
	**Being vaccinated with COVID-19**				
	Yes	Ref			
	No	**3.156**	**0.034**	**1.088**	**9.154**
	**Collecting bio-samples in the community**				
	Yes	**2.917**	**0.005**	**1.373**	**6.196**
	No	Ref			
	**Supporting vaccination spots**				
	Yes	**2.347**	**0.033**	**1.073**	**5.135**
	No	Ref			

^*^Odds ratio adjusted by age, sex.

## 4. Discussion

Our study provides insight into the depression experienced by nursing students in Vietnam, who voluntarily joined the nursing workforce on the frontline due to the rapid spread of the COVID-19 pandemic. During the pandemic, individuals were put under tremendous stressful conditions resulting in a higher risk of developing depression, particularly for nursing students on the frontline. The results of the present study revealed that 21.5% of nursing students suffered from mild to extremely severe depression. Our findings were consistent with the pooled prevalence rate of depression among healthcare workers during COVID-19 reported by Pappa et al. (27). Using the same DASS-21 depression scale, the percentage of risk of depression among nursing students in our study was similar to the depression rate of 26.8% in Chinese medical student volunteers, according to Zhang et al. (28). Likewise, another survey conducted among front-line healthcare workers in Italy found that 28.6% of healthcare workers experienced depression measured with the DASS-21 depression scale (29). However, in terms of targeted populations, healthcare workers who were nurses comprised only 25% of participants in that study. In comparison to a similar study conducted in Hanoi during the first wave of Covid-19 pandemic, the percentage of students who perceived depression in our study was higher than the prevalence of students who screened positive for depression, with 21.5 and 14.5%, respectively (11). The difference can be explained by the discrepancies in the depression scale. In addition, recent studies highlight that healthcare workers, including nurses who are directly engaged in the frontline, are more prone to mental disorders (30). Besides, the rate of depression in our results is lower than in another study conducted among Vietnamese students with the rate of depression being 46.0% (31). One possible explanation for this difference comes from the academic year of the sample, which indicates that the rate of depression among 1 or 2-year students is higher than the rate of depression among four or beyond 4-year students due to their stable mentality (31).

Interestingly, a significant proportion of the study participants (49.2%) did not use coping strategies during their working time on the frontline. However, our study findings explicated that the rest were using various strategies to cope with work-related mental health problems such as video consultations, joining an online support network, online consultations for individuals/groups, and receiving partial support from professionals. Nursing students spontaneously utilized these coping strategies without any instruction or guidelines before or during their frontline workforce participation. Therefore, those implemented tactics seemed not to address the depression they had, as our study found no statistically significant association between depression and coping strategies. This finding is like the previous work in Tohoku Region, Japan (32). In the previous research, various coping strategies were frequently used among nurses to manage their depression, such as a positive approach, problem-solving, and positive re-evaluation (33). Even though coping is a context-dependent phenomenon, coping strategies to avoid mental distress pre- and in-healthcare services supplies should be integrated into nursing education programs and into continuous training for vulnerable groups such as female students, or those at risk of mental health issues as a way to better prepare students for managing depression while responding to a crisis or health disasters in the future.

In addition to equip students with depression management training, the delivery method of training and support programs should meet the nursing students' preferences to be effective. This study highlighted a significant association between an online network and depression among nursing students (*p* < 0.05). This finding was consistent with one study from Nigeria that revealed emotional support was an effective coping strategy against depressive symptoms (34). Another survey conducted among healthcare workers in Vietnam shortly after the first wave of COVID-19 also confirmed that those with severe stress levels expected to receive psychological support from web-based interventions (18). Considering this evidence, developing a web-based mental wellness program to support nursing students working at the frontline during a health crisis is recommended.

Working non-stop days-and-nights, high risk for depression is understandable, and thus, depression risk factors should be managed to support nursing students stay-well and work-well. This study asserted that female students were more likely to experience depression than their male counterparts (AOR = 2.67, 95%CI 1.07–6.70). This finding is aligned with previous studies confirming a higher risk of developing depression and anxiety among women working in health workforces during a pandemic or crisis (30, 35–37), suggesting a focus on female groups while providing depression management programs for nursing students. In addition, we also found evidence that the risk of depression among nursing students who were not vaccinated against COVID-19 was three times higher than those who had been vaccinated. Protected with vaccination is therefore essential in increasing confidence and reducing depression for nursing students working in high-risk-of-infection environments.

This study found that nursing students who participated in collecting bio-samples in the community and providing support to vaccination spots had a higher risk of depression than nursing students who oversaw other tasks. It is understandable because having regular direct contact with suspected patients or their bio-samples increases the risk of exposure, leading to increased depression (35). As reported by previous research, the fear of direct contact with patients led many respondents to volunteer in non-patient contact works other than patient-contact activities.

We acknowledged that our study had a few limitations. First, we used an online survey for data collection, and therefore, we may have missed some of the potential nursing students willing to do this survey. Nonetheless, collecting data via an online platform was the only possible solution during the lockdown period, the advantage and convenience of this method promise a large application even when the COVID-19 situation is over. Second data collection was conducted in three provinces where considered as hotspots of COVID-19 explosion and where healthcare students were mobilized to support the local health staff. Controlling the setting of the study in three large provinces of Vietnam during the fourth way of COVID-19, our study could have some limitations in data generalizability. Finally, anonymous self-reported data could result in inaccurate data, despite the fact that we made an effort to validate the participants by carefully checking the answer of the respondents. Further studies on a larger scale should be conducted in the future to demonstrate the representative of nursing students in Vietnam.

To recapitulate, our research results have attempted to prove that the mental health of nursing students who were working on the frontline is affected by the COVID-19 pandemic and there was no reliable coping strategy for depression among those. Therefore, it is necessary to develop some feasible interventions to positively foster nursing students' mental health, especially in the case of working in quarantine areas.

## 5. Conclusion

In conclusion, this cross-sectional study indicated a high prevalence of mild to extremely severe depression among Vietnamese nursing students working on the frontline against COVID-19, which was 21.5%. Being females, collecting bio-samples, providing support to vaccination spots, and not vaccinating against COVID-19 were found as risk factors for depression among this population. Our findings revealed that depression management and coping strategies should therefore be integrated into nursing programs for nursing students at educational institutes to better prepare them for working in a crisis in the future. The future training programs should also focus on factors associated with depression as found by the study, and how nursing students can manage these factors while working in a high-risk depression environment.

## Data availability statement

The raw data supporting the conclusions of this article will be made available by the authors, without undue reservation.

## Author contributions

All authors listed have made a substantial, direct, and intellectual contribution to the work and approved it for publication.
